# Distrust as a form of inequality

**DOI:** 10.1038/s41598-023-36948-x

**Published:** 2023-06-19

**Authors:** Jennifer T. Kubota, Samuel A. Venezia, Richa Gautam, Andrea L. Wilhelm, Bradley D. Mattan, Jasmin Cloutier

**Affiliations:** 1 Department of Psychological and Brain Sciences, University of Delaware, 105 The Green, Newark, DE 19716 USA; 2 Vivid Seats, 24 E Washington St, Suite 900, Chicago, IL 60602 USA; 3 Department of Political Science and International Relations, University of Delaware, 18 Amstel Ave., Newark, DE 19716 USA

**Keywords:** Psychology, Human behaviour

## Abstract

Navigating social hierarchies is a ubiquitous aspect of human life. Social status shapes our thoughts, feelings, and actions toward others in various ways. However, it remains unclear how trust is conferred within hierarchies and how status-related cues are used when resources are on the line. This research fills this knowledge gap by examining how ascribed, consensus-based status appearance, and perceived status appearance impact investment decisions for high- and low-status partners during a Trust Game. In a series of pre-registered experiments, we examined the degree to which participants trusted unfamiliar others with financial investments when the only available information about that person was their socioeconomic status (SES). In Study 1, SES was ascribed. Studies 2 and 3 conveyed SES with visual antecedents (clothing). Across all three experiments, participants trusted high SES partners more than low SES partners. In addition, subjective perceptions of status based on visual cues were a stronger predictor of trust than consensus-based status judgments. This work highlights a high status-trust bias for decisions where an individual’s money is on the line. In addition, high-status trust bias may occur simply because of an individual’s subjective assumptions about another’s rank.

## Introduction

Social hierarchies are a systemic and intrinsic part of organizations^1^ and culture^2^. Hierarchies can have different foundations. For example, those based on financial status give higher rank to those with greater capital, those based on merit give higher rank to those with higher competence. These differences may also allow for varying levels of competition—ladder hierarchies are more zero-sum than pyramid hierarchies. However, all create a rank order for their members^3^. Thus, status is an organizing aspect of daily life, and as such, humans infer status from many cues and use this information to navigate social relationships.

Although status-based impressions and decisions have received less attention than other salient social categories such as gender, race, and age^4^, a growing literature has examined how attention and evaluations are shaped by perceived status. In this literature, high-status individuals are often perceived as more valuable, competent, and rewarding than low-status individuals^5–7^. Social status can be simply construed as the relative rank of an individual along one or more valued social dimensions within a given social hierarchy^6^. As a testament to the pervasive impact of status on our perception of others, even when not explicitly stated or known, hierarchical information inferred from actions^8^ and alliances^9,10^ has been shown to shape impression formation. Previous research has found that high-status individuals are attended to more and often conferred with positive traits and thereby evaluated more favorably^6,11–15^. For example, high-status others are associated with greater attractiveness^16^. Therefore, high status conspecifics tend to be preferentially attended to and evaluated.

However, status is not a monolith, and individuals only sometimes respond positively to those of higher status, depending on the dimension of status and context^6^. For example, when individuals formed impressions of male targets paired with knowledge of either their moral or financial status, they showed greater positive evaluative responses to moral (vs. immoral) and less wealthy (vs. wealthy) targets^13^. Additionally, although individuals described as upper-class can be perceived as more unethical or selfish^17,18^, individuals of high prestige status tend to be perceived as more ethical and trustworthy^19^. While people occupying lower status positions in the hierarchy may not typically control resources, they may be of help, assumed to be less greedy, or perceived as more cooperative. For one, lower status members may be more willing to share^20,21^. Most importantly, lower status members are more likely to be underserved, and our trust in them is more consequential. Therefore, individuals may be motivated to help lower status partners obtain financial gains^22^. Based on Mattan and colleagues' theoretical framework^6^ and this previous research, it remains unclear whether people will trust high- versus low-status individuals more in an interdependent context.

Because status differentially shapes evaluative responses depending on the context, it remains unclear whom people trust to cooperate and reciprocate trust? Is it someone of higher status who may possess relevant skills and control valuable resources^1,5,23–29^, but may opt not share them^30,31^? Or is it the person of lower status who may be perceived as less greedy, more empathic, and more willing to reciprocate to arrive at a mutually beneficial outcome^21,32,33^? More specifically, although social status fundamentally shapes how we think, feel, and behave towards others^6^, it remains unclear how trust is conferred within hierarchies based on socioeconomic status (SES, hereafter referred to as “status”)^34–37^ and what cues we use to infer status when trusting others with our resources. In the present research, we fill this gap in knowledge, by examining how ascribed (i.e., conveyed via cues paired with status knowledge) and perceived (i.e., inferred from visual cues) SES of individuals who are either high or low in this status dimension impacts financial investments during a resource sharing task called the Trust Game^38^.

Trust is a vital component of social relationships and can encourage cooperation^39–43^. Trust can be defined as a psychological state involving a willingness to expose oneself to vulnerability by having positive expectations regarding the intentions or actions of another individual^43^. The investment game is a common tool to assess trust because the amount a player can earn depends on another player’s decisions. During the Trust Game, mutually beneficial decisions depend on a partner’s assumed cooperativeness. An individual (investor) can earn additional money by trusting a partner (trustee), but if that player decides not to reciprocate, they risk losing all the money. Players can also choose not to invest in a partner and keep the money. Trusting in this game is risky because the outcome is highly uncertain^41,44,45^. If a player assumes that a partner will not reciprocate, the rational choice for a purely self-interested individual is to invest nothing. However, research consistently finds that cooperation is the norm among players of the Trust Game—the average investor typically sends a significant share of their endowment, and when an interaction partner is a real person, trustees usually reciprocate^46,47^.

When deciding whom to trust, individuals evaluate numerous factors, including how trustworthy someone appears and their attributes or past behaviors^48–54^. Status is no exception^6,55,56^; rank information is quickly extracted from distinct antecedents. For example, perceivers use visual cues (e.g., race, clothing, body posture, facial structure, and even car ownership) and knowledge about a target’s ascribed rank within that hierarchy to infer status and shape subsequent evaluations^6,57,58^. While we know that people are highly vigilant to status-relevant cues (e.g., physical appearance, behaviors) when making spontaneous judgments about whom to trust^59,60^, it is unclear which antecedents of status (i.e., perceptual (e.g., facial dominance, attire) or knowledge-based (e.g., prior knowledge of status through names or ascribed attributes)) are more likely to impact trust in a reciprocal investment context. More specifically, when deciding whom to trust, do ascriptions (objective knowledge) of status impact trust similarly to perceptual antecedents of status (assessments of status based on visual cues)?

Moreover, when given only visual antecedents of status, do humans rely on consensus of someone’s status based on appearance or instead rely on their subjective evaluation of another’s status appearance when deciding whom to trust? The consensus of the status appearance based on societal stereotypes (i.e., fancy jewelry, dominant face, haute couture clothing, etc.) may not reflect the status inferred via subjective perception of the whole person (i.e., someone is wearing expensive clothing, but not perceived to be high status). Though this subjective perception of rank may not be accurate, it can impact behavior^6,22^. According to Mattan and colleagues’ theoretical framework^6^, an individual’s subjective assessment of status (an individual’s evaluation of partner status) should be a better predictor of trust compared to consensus evaluations of status (status based on external societal evaluations) because the meaning and influence of status depends on the perceiver’s evaluation. Therefore, a second consideration in the current research is whether consensus ratings of status cues predict trust to the same degree as an individual’s subjective assessment of status.

### Current study

In this series of pre-registered experiments, we examined the degree to which participants trust unfamiliar others with financial investments as a function of their SES. In Study 1, SES was ascribed and ostensibly objective. In Studies 2 and 3, SES was conveyed with visual antecedents (clothing) and based on consensus ratings of the clothing. In Studies 2 and 3, participants also provided subjective status ratings for the targets at the end of the experiment. Based on previous work suggesting that individuals of higher SES tend to be evaluated more positively, we hypothesized that participants would trust high SES individuals more relative to low-status individuals both when SES knowledge is ascribed (Study 1) and when it is perceived via visual antecedents (Study 2 and 3). For Studies 2 and 3, we further hypothesized that subjective perceptions of status (and not just the consensus of the status of the attire) would impact trust, such that those who are subjectively perceived to be high status would be trusted more.

## Experiment 1

To examine how ascribed SES impacts trust decisions, in Study 1, participants played a single-shot Trust Game with partners who were either high or low in SES. We hypothesized that participants would invest more money (i.e., index of trust) in high versus low SES individuals (confirmatory). Additionally, previous research has found that individuals use previous feedback (i.e., how much a partner returned in the previous round) to inform their investment decisions in the current round, even when such feedback is not dependent (i.e., is from a totally different partner and should have no impact on responses to a new partner)^61^. In this study, we explored how the previous partner’s feedback and ascribed status impacted subsequent trust decisions as a function of the current partner’s status (exploratory).

## Results and discussion

**Figure 1:**
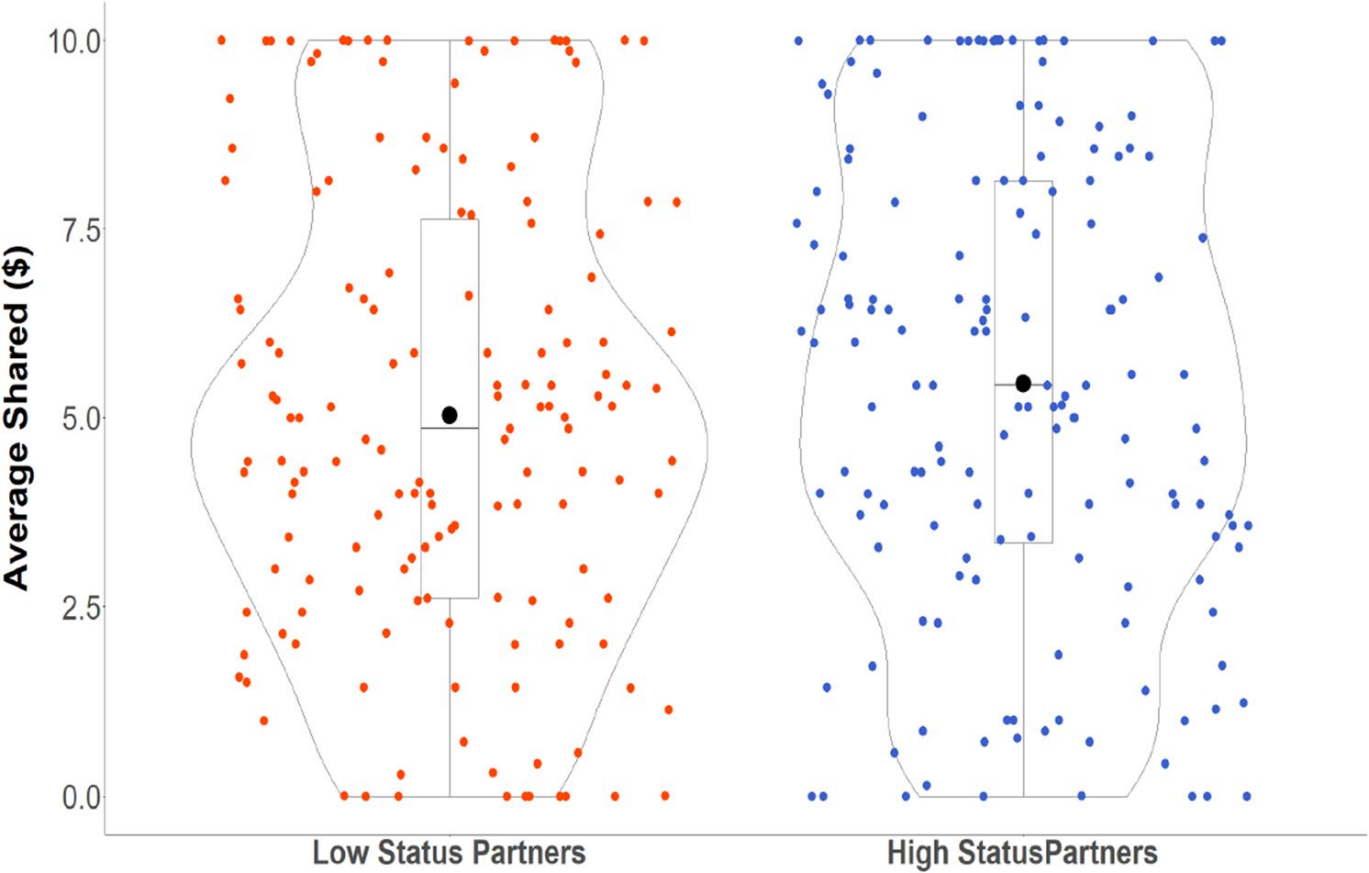
Trust decisions as a function of partner status. Each dot represents a participant’s data point (red dots for when participants played with low-status partners and blue dots for when participants played with high-status partners). The y-axis represents the amount of money participants invested in their partners (our measure of trust). The effect size and the 95% confidence interval are displayed as a point estimate and vertical bar (black dot and line, respectively). There was greater money trusted to high-status partners compared to low-status ones.

Supporting our confirmatory hypothesis, participants invested more in partners ascribed with high SES than partners ascribed with low SES, as confirmed by a significant main effect of partner’s SES, *b* = 0.322, *SE* = 0.061, *CI*_*95%*_ = [0.202, 0.443],* z*(7) = 5.259, *p* < 0.001 (see Fig. 1; generated in R^62^). However, we did not find that partner feedback (i.e., how much the partner returned in the previous round) significantly predicted trust decisions, *p* = 0.124, nor did partner feedback significantly interact with partner status, *p* = 0.266. The significant main effect of partner status predicting greater trust decisions was consistent with our pre-registered prediction. Participants trusted partners who were ascribed high status more than partners who were ascribed low status. However, it is possible that trusting high-status individuals does not generalize to situations where knowledge of status is not provided, but rather derived from a perceptual antecedent^6,63^. In the next experiment, we examined whether a more subtle and potentially ecologically valid form of status^61,64,65^ would have similar consequences for trust.

## Experiment 2

To examine how visual antecedents of SES based on clothing impacts trust decisions, in Study 2 participants again played a single-shot Trust Game with partners who were wearing clothing that were pre-rated as either high or low in SES. In addition, we included an exploratory measure of subjective SES appearance ratings of each partner that participants provided following the Trust Game. We hypothesized that participants would invest more money (i.e., index of trust) in high- versus low-SES individuals as a function of the consensus SES ratings (confirmatory) and that subjective ratings of SES appearance would also predict trust (exploratory).

## Results and discussion

In Experiment 2, participants completed the Trust Game with partners who were assigned to the low-status or the high-status group via consensus ratings of attire. In contrast with results from Experiment 1 and our pre-registered prediction, participants did not significantly differ in the extent to which they invested in partners dressed in high-SES (vs. low-SES) attire, *b* = 0.020, *SE* = 0.079, *CI*_*95%*_ = [−0.136, 0.175],* z*(18) = 0.247, *p* = 0.805. However, unlike Study 1, participants did show a significant tendency to invest less in their current partner if their partner on the previous trial shared rather than kept their investment from the participant, *b* = -0.210, *SE* = 0.097, *CI*_*95%*_ = [−0.399, −0.020], *z*(18) = −2.165, *p* = 0.030. This effect did not significantly depend on the status of the current partner’s attire: Status × Feedback interaction, *b* = −0.134, *SE* = 0.158, *CI*_*95%*_ = [−0.444, 0.177], *z*(18) = −0.843, *p* = 0.399.

**Figure 2:**
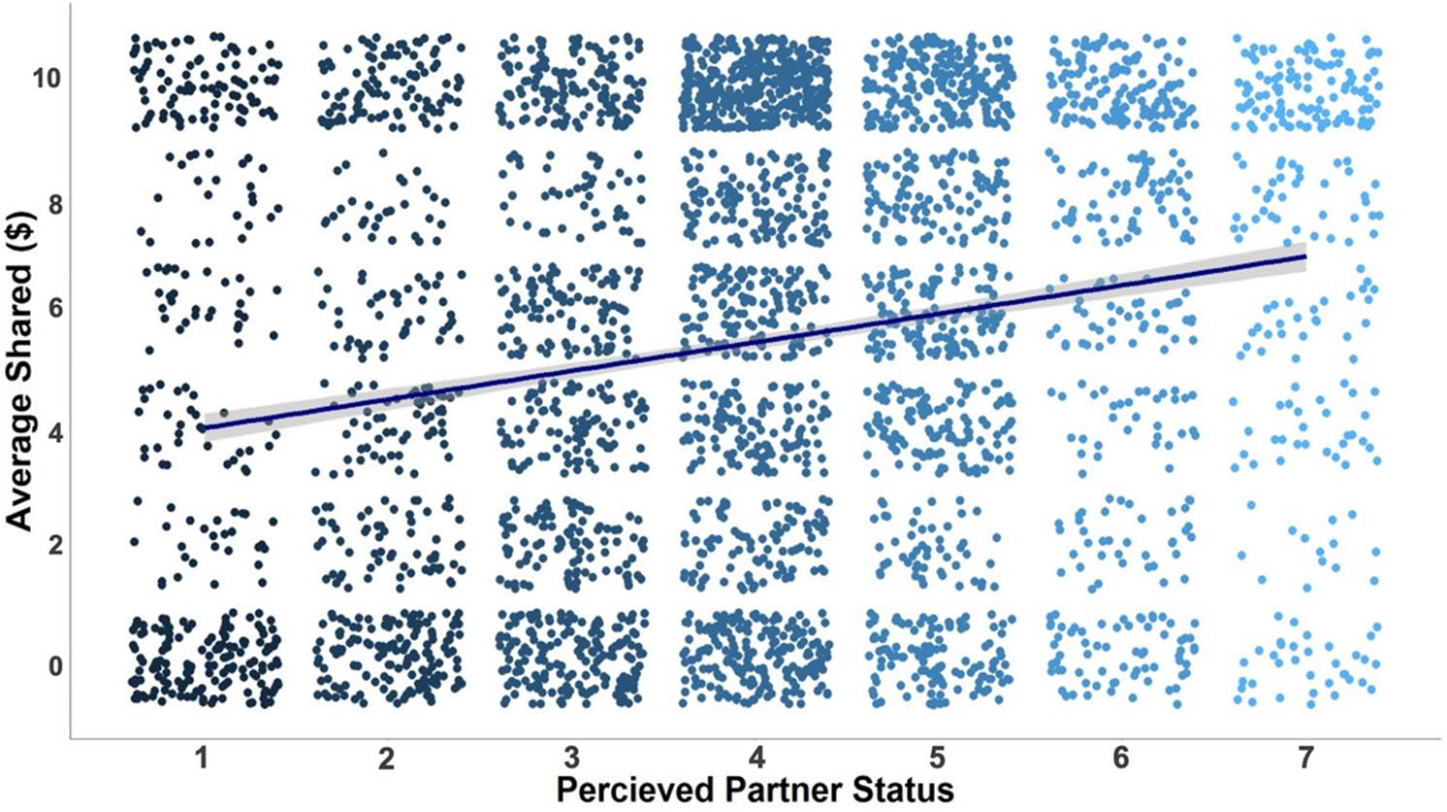
Subjective status ratings of partners predicted trust. The y-axis represents the amount of money participants invested in their partners (our measures of trust). The x-axis is the participants subjective ratings of the partners’ status. Each blue dot represents a participant’s decision. The line represents the linear effect with the confidence interval around that estimate. Participants trusted partners more as their subjective perceptions of social status increased.

Exploratory analyses used each participant’s post-game ratings of the perceived social status of each face as an alternative predictor in lieu of our a priori categorizations of target status based on pre-rated attire status. Results from these analyses showed that participants invested more in partners subsequently rated as higher in social status compared to partners rated as lower in social status, as indicated by a significant main effect of post-game perceived partner status, *b* = 0.279, *SE* = 0.059, *CI*_*95%*_ = [0.163, 0.396],* z*(9) = 4.717, *p* < 0.001 (see Fig. 2; generated in R^62^). Because participants’ ratings of perceived status were measured only after the Trust Game, it is possible it was influenced by whether the partner reciprocated during the trust game. However, although exploratory analyses suggested that partner reciprocity (vs. defection) did predict significantly greater post-game perceived trustworthiness, *b* = 0.271, *SE* = 0.066, *CI*_*95%*_ = [0.141, 0.401],* z*(8) = 4.078, *p* < 0.001, these same in-game outcomes failed to significantly predict post-game perceived social status, *b* = 0.096, *SE* = 0.066, *CI*_*95%*_ = [−0.033, 0.225],* z*(8) = 1.456, *p* = 0.145.

Experiment 2 provides some additional support for the hypothesized impact of status on trust, with individuals trusting partners perceived to be high status more than those perceived to be of low status. This occurred even despite the amount previous partners returned (i.e., feedback). These results also suggest that there may be important individual differences in how visual antecedents of status are perceived. When considering consensus ratings of status based on ratings made by previous participants, no difference in trust was observed. However, participant’s subjective ratings of the status of each partner did relate to how much they trusted them. Specifically, the greater a participant’s subjective perception of partner status, the more participants trusted them during the game.

## Experiment 3

Study 3 aimed to replicate the exploratory finding from Study 2 that participants’ idiosyncratic ratings of perceived status appearance shape their trust decisions using a larger and well-powered sample. Participants again played a single-shot Trust Game with partners who were wearing clothing that were pre-rated as either high or low in SES. We again assessed participants’ subjective assessments of each partner’s SES appearance. We hypothesized that subjective ratings of SES appearance would predict trust ratings (confirmatory), such that participants perceiving partners as higher status would also trust them more.

## Results and discussion

**Figure 3:**
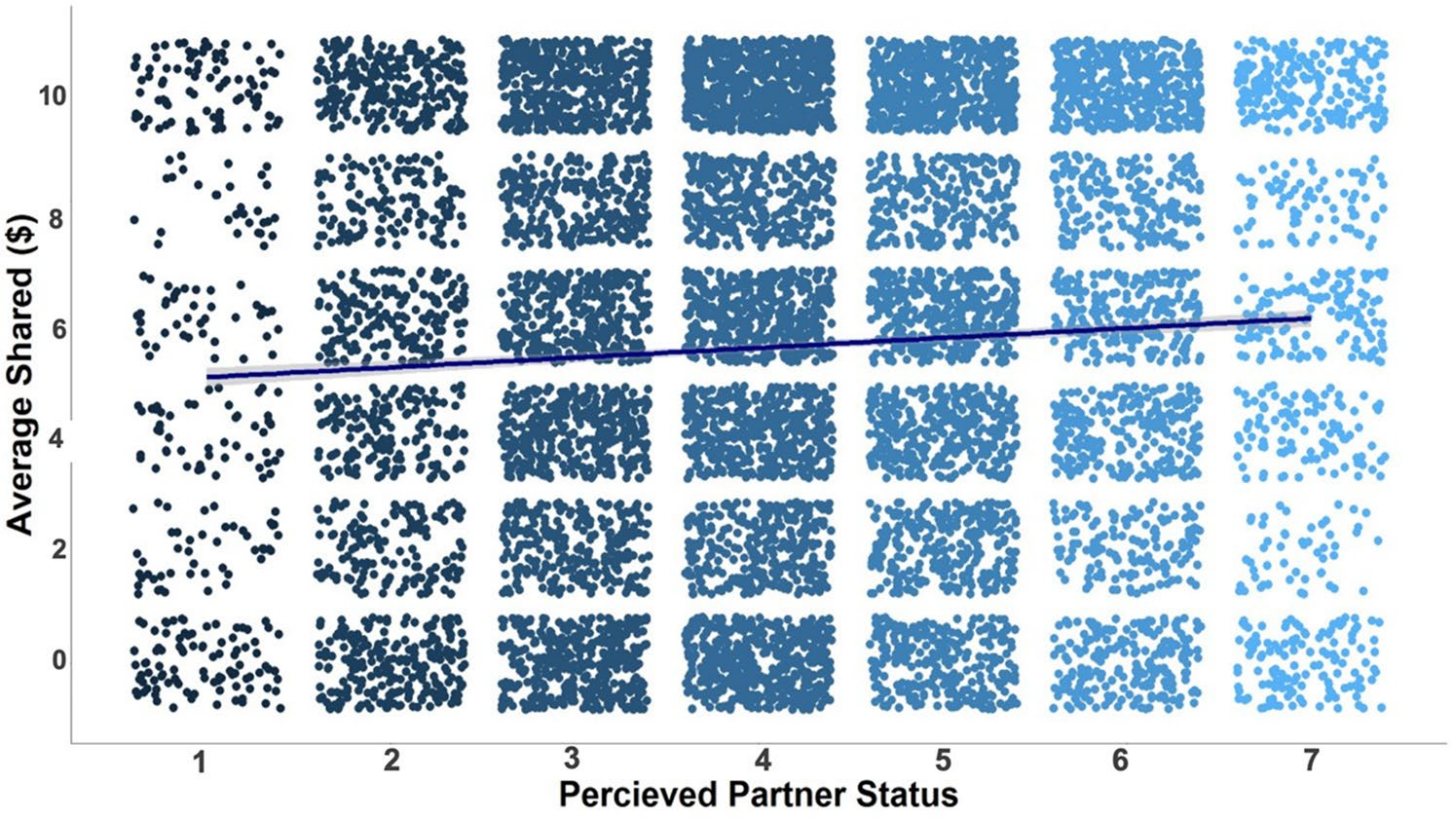
Subjective status ratings of partners predicted trust. The y-axis represents the amount of money participants invested in their partners (our measures of trust). The x-axis is the participants subjective ratings of the partners’ status. Each blue dot represents a participant’s decision. The line represents the linear effect with the confidence interval around that estimate. Participants trusted partners more as their subjective perceptions of social status increased.

In our final study, results of subjective status ratings of the partners replicated Experiment 2. Once again, social status selectively impacted trust. Participants invested more in partners whom they later rated as higher in SES compared to partners who they later rated as lower in SES, as indicated by a significant main effect of perceived partner SES, *b* = 0.135, *SE* = 0.022, *CI*_*95%*_ = [0.092, 0.178],* z*(7) = 6.156, *p* < 0.001 (see Fig. 3; generated in R^62^). This finding was consistent with our pre-registered prediction and our exploratory findings from Experiment 2. The effect of perceived status on trust decisions remained significant even after controlling for the previous trial’s outcome, which, similar to Experiment 1, did not produce any significant effects, *p* > 0.18 (cf. Experiment 2).

Planned exploratory analyses also showed that partners who reciprocated (vs. defected) were later rated after the Trust Game as higher in trustworthiness and likeability, *b* = 0.157, *SE* = 0.040, *CI*_*95%*_ = [0.079, 0.235], *z*(9) = 3.957, *p* < 0.001, and *b* = 0.096, *SE* = 0.040, *CI*_*95%*_ = [0.019, 0.174],* z* = 2.429, *p* = 0.015, respectively. In-game outcomes did not significantly predict post-game perceived status or dominance, *b* = 0.042, *SE* = 0.039, *CI*_*95%*_ = [−0.035, 0.118], *z*(8) = 1.064, *p* = 0.287, and *b* = −0.039, *SE* = 0.039, *CI*_*95%*_ = [−0.116, 0.037],* z*(8) = −1.009, *p* = 0.313, respectively. These findings were consistent with Experiment 2, where we observed that reciprocating (vs. defecting) partners were rated subsequently as more trustworthy, but not significantly higher in social status.

**Figure 4:**
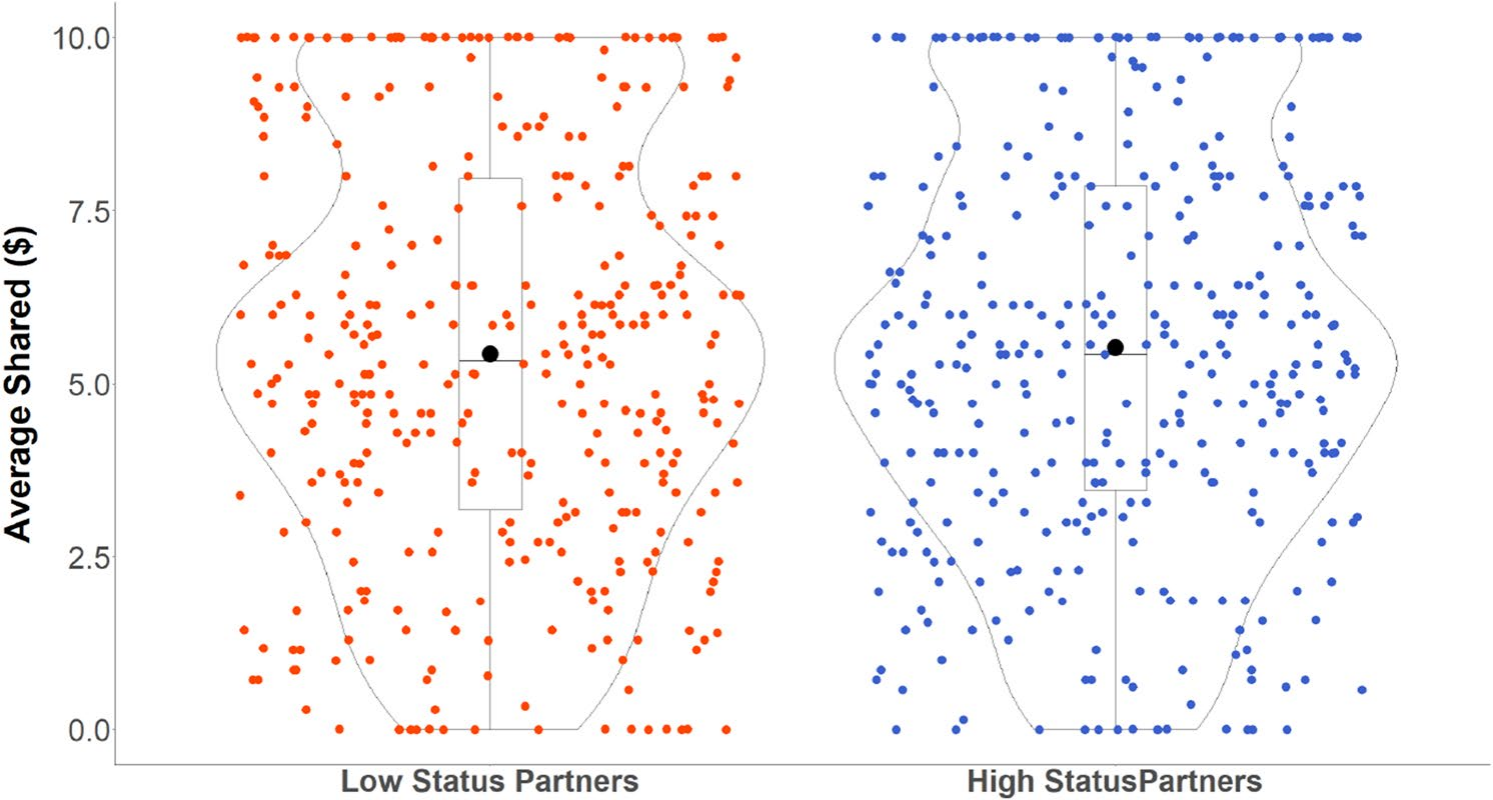
Trust decisions as a function of consensus ratings of the attire status. Each dot represents a participants’ data point (red dots for when participants played with partners wearing low-status attire and blue dots for when participants played with partners wearing high-status attire). The y-axis represents the amount of money participants invested in their partners (our measures of trust). The effect size and the 95% confidence interval are displayed as a point estimate and vertical bar (black dot and line, respectively) There was greater money trusted to partners wearing high-status attire compared to low-status attire.

Unlike Experiment 2, exploratory analyses revealed that participants also invested more in partners who were wearing high-SES (vs. low-SES) attire, as indicated by a main effect of prior independent ratings of attire status (consensus ratings), *b* = 0.093, *SE* = 0.039, *CI*_*95%*_ = [0.015, 0.170],* z*(7) = 2.347, *p* = 0.019 (see Fig. 4; generated in R^62^). This finding was consistent with our pre-registered prediction for Experiment 2, which was not ultimately supported in that experiment with fewer participants. This effect of prior ratings of attire status in Experiment 3 remained significant even after controlling for the previous trial’s outcome, which did not have any significant effects in this exploratory analysis, *p* > 0.14. Therefore, in a more highly powered experiment, both consensus ratings of status based on perceptual cues (Fig. 4) and participants’ subjective ratings of their partner’s status (Fig. 3) shaped trust. However, when examining the effect sizes, subjective assessments of higher status based on appearance was related to increased trust (14.45% increase) compared to consensus ratings of higher status (9.75% increase). These findings suggest that individual subjective assessments of status might more strongly predict trust when relying on perceptual cues.

## General discussion

Findings from the current pre-registered and highly-powered research demonstrates that social status, a ubiquitous feature of organizations and other hierarchical social structures, impacts decisions to trust. Importantly, status shapes trust decisions towards people both when their status is known and when it’s inferred based on appearance. In three experiments, in which status was manipulated based on different antecedents (knowledge or perceptual cues), we consistently found greater trust towards high-status partners. Indeed, this was observed both when status was ascribed based on knowledge or pre-existent consensus ratings of appearance (Experiments 1 and 3) and when it was subjectively inferred from appearance by participants (Experiments 2 and 3). Combined, these findings extend our previous theoretical framework suggesting that status fundamentally shapes our evaluations of others and our trust decisions^6,58^.

In addition, this research highlights the importance of differentiating how distinct antecedents of socioeconomic differentiation influence trust. SES is a multidimensional construct usually based on objectively assessed dimensions such as income, occupation, and education level^6^. This construct is a frequently utilized dimension of social status for humans and used in a variety of contexts individuals are ranked based on their status^6^. Prior work has explored how status manipulated via knowledge (e.g., university affiliation, performance rankings, provided SES, managers/employees) shapes trust decisions^34–36^, but no work to our knowledge has examined how perceptual cues of status impact trust. These findings also highlight how an individual’s subjective evaluation of another’s status impacts trust. High-status trust bias may occur simply because of an individual’s assumptions about rank based on appearance^66^. These results highlight the implication of status appearance for first impressions of trust during everyday encounters between individuals that lack explicit knowledge of each other’s status.

These findings also underscore that subjective evaluations of status appearance can be a stronger predictor of trust than consensus ratings of status based on appearance. Specifically, in Study 3 when examining the effect sizes, subjective assessments of status were associated with a greater increase in trust (14.45% increase) than status based on consensus of ratings of appearance, which was associated with a smaller increase in trust (9.75% increase). Moreover, in Study 2, which had lower power, subjective status ratings of appearance, but not the consensus ratings, predicted trust. Together these findings highlight the importance of individual idiosyncrasies when investigating status inferences. If a person believes that skinny jeans are a symbol of high-status (and maybe they are in a particular social circle) but, on average, folks think skinny jeans are a low-status cue (i.e., the consensus is that skinny jeans are low-status attire), personal subjective ranking may weigh more heavily on trust decisions than consensus. The relative contribution of subjective versus consensus evaluations of the status of perceptual cues should be further examined because the impact of these perceptual cues may be more subtle and individually defined.

Importantly, status impacted trust decisions irrespective of if others reciprocated. Although we found that people trust partners more if they reciprocate^67^, reciprocation across a game did not impact the relationship between status and trust decisions in two of three studies (cf. Experiment 2). This result is largely consistent with previous research suggesting that status alters expectations of another’s benevolence intentions^36^, and the current findings highlights that this occurs independent of how others are behaving during the game.

Another consideration is the relationship between power and status. Mattan and colleagues^6^ have discussed how status and power are distinct. Although power plays an important role in dominance-based status acquisition and maintenance, we caution against equating dominance with power. Power implies a degree of control over material or social resources^26^. However, control over resources may not necessarily be used to enhance one’s own status. Individuals preferring a prestige-based approach to acquiring status may, in fact, avoid using their power to enhance or preserve status if it would be perceived as overt coercion or manipulation^28,30,68^. In these experiments, power and status were orthogonal as both players had power to determine the outcomes of the game because of the interdependent nature of the task. In addition, faces were equated on perceived dominance, and in game behavior did not predict subsequent dominance judgments. Therefore, we do not think differences in dominance or power can explain our results, but we view this as an interesting future direction.

Given previous research demonstrating that individuals view upper-class people as more unethical than lower-class people^18,69,70^, it may seem surprising that individuals trust high-status partners more than low-status partners. Indeed, previous research found a tendency for third-party arbitrators to preferentially punish privileged others who make selfish decisions^22^. However, even if individuals at times view high-status others as unethical^19^, when their own money is on the line, they may be motivated to trust them. This would suggest a distinction between decisions involving high-status others from a third-party perspective (i.e., when making decisions on behalf of others) and decisions involving high-status others from a first-person perspective (i.e., when the decisions directly impact the individual). One explanation for this is that, on average, perceivers view high-status others as more competent^71–73^ and expect positive interactions with them^74^. Future research should directly compare first- versus third-person status-based decision-making.

### Future directions

Although trust can increase cooperation and performance^39,75,76^, trusting others solely based on their known or perceived status may be misplaced. Mistrust can be costly. Because perceptual antecedents of status are varied and, at times, culturally- or context-dependent, it will be essential to consider further the cues and contexts where such bias can be amplified. Moreover, consideration should be given to how these perceptual signals of status (e.g., faces, body language, attire, cars) confer trust cross-culturally^58^. Therefore, future research should consider the contexts that amplify status-based trust biases and their implications for potential discriminatory behavior.

It is also yet unknown if trust bias in favor of high-status individuals depend on the social dimension conveying trust in a given hierarchy. Previous behavioral and neuroimaging research found that evaluations of high-status individuals vary depending on the dimensions of status (e.g., moral versus financial status)^11,13^ or during times of conflict^77^. Because evaluations shape social judgements, it is possible that different dimensions of status lead to different biases in trust.

The importance of various dimensions of status as well as the stability of hierarchies can vary cross-culturally^6^. Therefore, culture represents a powerful context that needs to be considered when studying status dynamics. For example, in the U.S., more agentic people may generally be seen as more powerful^78^. However, in Japan, people who know more may be considered more influential^79^. Culture may also vary in how status is allocated among individuals or groups, as well as the degree of acceptance of this inequality^80,81^. Consequently, trust levels within a group (e.g., within a lower-status group) may be considerably higher compared to trust levels between groups (lower- and higher-status groups). Therefore, findings from this research may only generalize to some cultures, and future research should investigate cultural variations in status-based trust bias.

Our results show how SES influences trust decisions during a unique financial exchange. However, many interactions are iterative. At each iteration, people gain knowledge about the behavior of others. This knowledge may violate or support expectations, notably expectations based on the partners’ SES. Violations of expectations have been found to drastically shape impressions^48,82–86^. When a partner’s reputation is the only information available, decisions to cooperate change as the partner’s reputation evolves over iterative interactions^39^. Accordingly, it would be important to explore how violations of status-based expectations impact social evaluations and trust decisions. These studies may reveal dynamic adjustments of trust decisions when individuals obtain knowledge to update their pre-existent expectations.

Status is at times inferred based on other’s perceived social group of belonging (e.g., race, gender). For example, race and socioeconomic status are often stereoyptically associated, as such measured associations of “Black people” with “poor people” are stronger than associations of “White people” with “poor people”^87–89^. Similarly, gender is often stereoyptically associated with competency status, as men are more strongly associated with competence than women^90–92^. Future work should assess how additional social dimensions or intersectional identities impact status biases in trust.

In the future, researchers should also consider additional factors of status that may not be encompassed in an economic trust exchange. For example, low-SES individuals are often expected to be under stress, resource deprived, or socially ostracized. These perceptions might lead low-status individuals’ choices to appear riskier to others. High-SES individuals, on the other hand, are expected to be competent, resourceful, and social, which might, for example, lead their decisions to share less to appear more selfish^6^. Manipulating these expectations may reduce status-based biases. For example, highlighting stereotypes related to warmth or honesty for low-status individuals may activate more favorable evaluations and increase trust for low-status others^93^. Exploring these different components of status-based trust may be fruitful future directions for bias reduction interventions.

Trusting others is an integral part of successful social interaction, and successful social interactions build good relationships. Therefore, trust decisions are often the first domino in a cascade of actions that result in forming social networks and advancing (certain) people within them. Therefore, future work must explore what drives greater trust biases towards higher status individuals and investigate ways to reduce the inequitable biases that may arise.

## Experiment 1 methods

Experiment 1 was pre-registered at https://osf.io/pge3c. All data and materials are publicly available at https://osf.io/tcjw5/.

### Participants

To determine our sample size, we conducted an a priori power analysis using the PANGEA application v0.2^94^. Power analyses suggested the sample of 147 was powered to detect effects of interest at above 80% (Supplemental Materials S1.1). To account for participant exclusion based on our a priori criteria (see Supplemental Materials S1.1), we aimed to oversample by 20 participants. However, because it can be difficult to precisely regulate the number of participants collected through Amazon Mechanical Turk (Mturk), we ultimately oversampled by 22 participants.

After implementing all exclusion criteria, the final sample consisted of 155 participants (74 Female, 80 Male, 1 Transmasculine). Participant ages ranged from 19 to 38 years old (*M* = 29.1, *SD* = 4.63). All participants were based in the United States and self-reported proficiency in the English language. One hundred and eleven (71.6%) participants identified as White, 20 (12.9%) as Asian, 14 (9.0%) as Black/African American, 5 (3.2%) as Latino/Hispanic American, and 5 (3.2%) as Biracial/Multiracial. Subjective SES scores of the participants ranged from 1 (lowest possible SES) to 9 (second highest possible SES), with an average subjective SES score of 4.76 (*SD* = 1.62). This study is in accordance with relevant guidelines and regulations. Participants provided informed consent in accordance with the guidelines set by the Declaration of Helsinki, and the experimental protocol was approved by the University of Chicago’s Institutional Review Board.

### Stimuli

Faces from 28 perceived White men from the Chicago Face Database^95^ served as stimuli for the Trust Game. Selected images were equated on contrast and luminance using the SHINE toolbox^96^, and backgrounds were changed to light gray. The resulting gray-scale images were cropped such that each contained only a face presented centrally on a 504 × 632 pixel frame. Faces were equated on various dimensions including likability, attractiveness, dominance, and trustworthiness (Supplemental Materials S1.1). In each of eight counterbalanced versions of the Trust Game, each of the four groups of faces were assigned to one Trust Game behavior (i.e., reciprocate, defect) and level of social status (i.e., high or low) based on colored borders (i.e., blue or orange). Each participant completed one of these eight versions of the experiment.

Previous research has relied on various antecedents of status including clothing, ascribed occupation/income/rank, body posture, facial structure, and even car ownership^6^. Although such cues may afford ecological validity in some contexts, these status cues do not always unambiguously convey status level. Moreover, perceptual antecedents of status such as clothing are demonstrably confounded with important social dimensions like competence^61,87^, making it difficult to reliably isolate effects of status. To avoid these potential pitfalls in our initial exploration of how status shapes financial trust decisions, we adopted a pre-existing procedure to ascribe status levels through learned color-coded background cues^13,22,55,97,98^.

### Procedure

Following a demographic screening survey and online consent form, participants began the experiment by completing a brief social status training task where they learned to associate two colors (blue or orange) with either high or low social status. After the status-color association training task, participants were introduced to the Trust Game. Prior to completing the Trust Game, participants took a brief 5-item quiz that assessed whether they understood the Trust Game. Participants then completed a 28-trial Trust Game. Both the SES–color association training and the subsequent Trust Game were presented online via Inquisit 5 Web Version 5.0.12^99^. Following the Trust Game, participants completed several exploratory behavioral questionnaires (see Supplemental Materials S1.1). After completing these additional measures, participants were then debriefed, given the option to exclude their data from analysis, and compensated.

### Tasks and measures

#### Learning status-color associations

Prior to completing the Trust Game, participants first learned to associate the colors blue and orange with different levels of SES (i.e., high or low). The status-color association training task began with the following definitions of SES used in previous research^55^: “*As you may know, those who are HIGH STATUS tend to be wealthy and university-educated, typically working in ‘white collar’ positions. As you may know, those who are LOW STATUS tend to be poor and high-school-educated (or less), typically working in ‘blue collar’ positions or unemployed*.” After reading these definitions, participants passively viewed a series of 12 images of darkened silhouettes over a colored background (i.e., orange or blue) paired with a sentence describing the silhouette’s color-specific SES level (six trials per SES level). The color–status pairings were counterbalanced between subjects. Next, participants completed a block of 36 trials in which they viewed the same color-framed silhouettes without ascribed SES information (other than the colored background) and responded to a prompt regarding the silhouette’s SES level (e.g., “Does this color mean HIGH or LOW status in the US?”). Participants had unlimited time to press 1 for high SES or 2 for low SES. Incorrect responses elicited an error message: “INCORRECT—please give the correct response in order to proceed”. At the end of the block, participants received feedback on their overall accuracy and instructions that they would repeat the preceding block, irrespective of their initial accuracy. Any errors resulted in repetition of this training block. Training concluded with the next successful completion of the training block at 100% accuracy^13,97,98^. On average, participants needed 1.26 blocks of 36 trials to reach 100% accuracy, consistent with previous work^22^. Although the present study did not test recall of status–color associations at the end of the experiment, previous research using this training procedure with relatively long cognitive tasks has shown good retention (~ 89%) and few differences when analyses were conducted with or without participants failing the post-task manipulation check^55^.

#### Trust game

**Figure 5:**
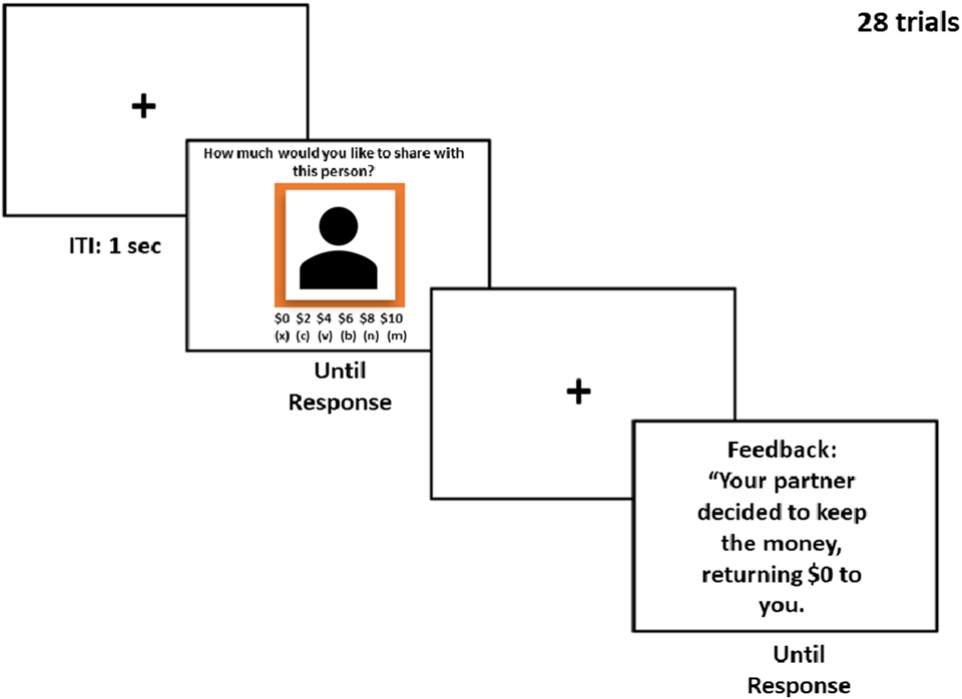
Trial sequence for the Trust Game. After a 1-s fixation, participants viewed a picture of their partner on top of a color background indicative of the partner’s status (low or high). Participants would see a face rather than a silhouette as pictured here. Participants then decided how much to share with this partner. After a 4-s blank screen, participants received feedback regarding their partner’s decision.

After completing the status training task, participants received instructions regarding the Trust Game (see Fig. 5; created in PowerPoint^100^). At the start of the Trust Game, participants learned that they would have the opportunity to make money by participating in economic interactions with different partners. During each interaction, participants received an allocation of $10. For every interaction with a partner, participants chose how much of $10 to share with their partner, in increments of $2 from $0 to $10. The participant kept any money that they did not share. The sum of any money that was shared with the partner was automatically quadrupled. The participant’s partner then had the option to either share back half of that quadrupled amount or keep all of it and return nothing to the participant who shared it with them. Participants were told that the partners were real people who had previously participated in the study in the opposite role as the participant. To increase the participant’s stake in the game, participants learned that one of their interactions would be randomly chosen at the end of the experiment to compute their bonus^38,61^. Specifically, the amount of money that the participant ended with on that randomly selected interaction would be divided by 10 and added to their Mturk compensation. In reality, each partner’s response was pre-programmed, with a 50/50 ratio of share and keep outcomes.

The Trust Game followed a within-participant design, such that each participant interacted with partners from each of the four conditions over a total of 28 trials, with 7 trials per condition: high status/share, high status/keep, low status/share, low status/keep. All four conditions were presented in a random order. Furthermore, the stimulus pairings were counterbalanced between participants such that each of the 28 partner faces appeared equally often as high or low status, and with share or keep outcomes across participants. Colored backgrounds were counterbalanced across status level across participants.

After reading the initial Trust Game instructions, participants responded to a set of five quiz questions that tested their understanding of the task instructions. Participants who responded incorrectly for four or more of the questions were excluded from analysis. After the quiz, participants also completed 4 practice trials that were identical to the experimental trials. Practice trials did not include faces appearing in the main task. Participants then completed 28 trials of the trust game with 28 unique partners. On each trial, participants made their decisions to share using the x, c, v, b, n, and m keys on their keyboard to choose amounts of $0, $2, $4, $6, $8, and $10 respectively. After participants made their decisions, they were presented a feedback screen indicating whether their partner reciprocated. If participants decided to share $0, they only received feedback indicating that they did not share any money during the trial.

Finally, we tested participants on their knowledge of status–color associations throughout the entire experiment. Participants learned in the preliminary instructions that there would be comprehension checks interspersed throughout the experiment. The first status–color association check occurred immediately followed the five quiz questions that preceded the Trust Game. The second check was prompted after the 10th Trust Game trial, and the third check was prompted after the 20th Trust Game trial. During these status–color association checks, participants viewed a silhouette superimposed on top of either a blue or orange background. As in the status–color association training, participants indicated whether the color shown indicated a high-status or low-status individual. Participants who failed at least 2 out of 3 of these catch trials were excluded from analyses.

#### Surveys

A series of exploratory self-report measures followed the Trust Game. In separate blocks, participants responded to items assessing (1) the perceived trustworthiness of each partner encountered during the Trust Game, (2) perceptions of whether high- or low-status partners shared back more, (3) the participant’s subjective and objective socioeconomic status, (4) social value orientation (SVO), and (5) general demographic information. After completing these measures, participants were debriefed and thanked for their participation. After debriefing, participants indicated whether they wished to remove their data from analysis. We report more information on each of these post-game measures below. Finally, participants received instructions about how to claim their base credit and receive their financial monetary bonus via Mturk.

#### Subjective status

Subjective socioeconomic status (SES) was assessed using the MacArthur Scale of Subjective Social Status^91^. The MacArthur scale presents participants with a ladder comprising rungs labeled 1 to 10 in ascending order, where a 1 represents the rank of people who have the lowest standing amongst the general population in the U.S. and a 10 represents the rank of people who have the highest standing among the general population of the U.S.

#### Objective status

Following previous recommendations^101^ and previous work exploring how perceived social status impacts decision making^22^, we collected single-item measures of participant income, education, and assets. These measures were used to compute a composite score of objective SES that equally weights income, education, and assets (see Supplemental Materials S1.1 for details).

#### Social value orientation

Social value orientation was measured through the 12-item Delaware adaptation of Liebrand’s Ring Measure^102^. The measure has 12 items, each of which has a unique resource allocation choice. Participants were told that they were randomly paired with another partner, and that they would make choices that could produce points for or take points away from themselves and their partner. Each of the 12 items has a different combination of two outcomes that the participant must choose between. By utilizing each of the 12 choices that the participant made, a categorical measure of social value orientation was computed for each person^103,104^.

#### Data analysis

The ordinal package^105^ in the R programming language^62^ was used to run all cumulative link mixed models. In our original pre-registration (https://osf.io/pge3c), we proposed using linear mixed-effects models with the lme4 package^106^. However, for the final experiment, we switched to using cumulative link mixed models because these models better account for non-continuous dependent variables like the discrete trust amounts used in the present research^105^. For the sake of consistency across experiments, we report cumulative link mixed models in the main text. However, linear mixed-effects models are reported for all experiments in the Supplemental Materials (see S1.2). Tests of our confirmatory hypothesis were the same regardless of analysis method.

For all analyses, we excluded trust responses from trials with RTs below 75 ms or over 10 s. To test our confirmatory hypothesis that higher ascribed status would predict greater trust, we entered partner status (low = -0.5, high = 0.5) as a fixed effect predictor of trust decisions in a cumulative link mixed model. For the exploratory analyses, we also added fixed effects for the previous trial’s outcome (partner kept = 0.5, participant did not share = 0, partner shared = -0.5) and the previous partner’s status (low = -0.5, high = 0.5), including the predictor for the partner’s status on the current trial and all possible interactions. For significant interactions, follow-up models were conducted to test simple effects. To further account for participant-specific variance, we allowed for between-participant variance in intercepts and slopes (i.e., random effects). In all analyses, we attempted to model as many random effects as possible without overfitting these data. In the event of convergence failures or model overfitting, we followed a uniform procedure for the simplification of random-effects structures (see Supplemental Material S1.3). Model syntax and contrast coding for all analyses are reported in the Supplemental Materials S1.3. The threshold for significance for all experiments was set at *p* < 0.05.

## Experiment 2 Methods

Experiment 2 was pre-registered at https://osf.io/s9cq6/. All data and materials for this study are publicly available at https://osf.io/tcjw5/.

### Participants

To determine whether the experiment was sufficiently powered, we again conducted an a priori power analysis for linear mixed models using the PANGEA application v0.2^94^. Results suggested a sample of 143 participants would be adequately powered to detect this main effect as small as d = 0.2 at 80.2% power. To account for exclusion of participants based on our a priori criteria, we planned to oversample by 20 participants, up to 163. US-based participants between the ages of 18–35 were recruited via Amazon Mechanical Turk (n = 163). Participants were required to have at least an 85% approval rating and at least 1000 completed tasks on Mturk to be eligible. Stricter inclusion criteria were used in Experiment 2 due to the emergence of data quality issues in Mturk samples in general that were discovered after Experiment 1 was conducted^107^. Participants meeting these basic inclusion criteria were further screened for eligibility via an attention and English proficiency test available through CloudResearch (then TurkPrime) prior to the main study. All participants were paid $2.00 for completing the full study, with an additional bonus varying between $0–2 USD, depending on a randomly selected trial in the study. To ensure data quality, we excluded from analysis 20 participants who failed at least 4 out of 5 questions on an initial instruction quiz that assessed whether participants understood the task. Lastly, we excluded from analysis 37 trials with response times slower than 10 s and 38 trials with response times faster than 75 ms. After implementing all exclusion criteria, the final sample consisted of 143 participants (73 Female, 69 Male, 1 Other). Participant ages ranged from 19 to 35 years old (M = 29.2, SD = 3.62). All participants were based in the United States and self-reported proficiency in the English language. Eighty-eight (61.5%) participants identified as White/Euro American, 19 (13.3%) as Asian/Asian American, 18 (12.6%) as Black/African American, 11 (7.7%) as Latino/Hispanic American, 5 (3.5%) as Biracial/Multiracial, 1 (0.7%) Native American, and 1 (0.7%) Middle Eastern/Arab American. Subjective SES scores of the participants ranged from 1 (lowest possible SES) to 8 (third highest possible SES), with an average subjective SES score of 4.82 (SD = 1.63). This study is in accordance with relevant guidelines and regulations. Participants provided informed consent in accordance with the guidelines set by the Declaration of Helsinki, and the experimental protocol was approved by the University of Delaware’s Institutional Review Board.

### Stimuli

To convey different levels of social status, we selected 28 faces of perceived White men from the Chicago Face Database^95^. Faces were equated on various dimensions including likability, attractiveness, dominance, and trustworthiness (Supplemental Materials S1.1). In each of eight counterbalanced versions of the Trust Game, each of the four groups of faces were assigned to one Trust Game behavior (i.e., reciprocate, defect) and level of social status (i.e., high or low) based on consensus ratings of the attire. Each participant completed one of these eight versions of the experiment.

To convey different levels of social status, we selected a set of 28 contemporary men’s attire that were developed in previous work based on images pulled from online clothing retailers^66^. Using independent prior ratings of the attire (*n* = 24–32), we were able to verify that high-status attire (*M* = 5.57, *SD* = 0.71) were rated as significantly higher in perceived status compared to low-status attire (*M* = 3.46, *SD* = 0.17), *t*_*Welch*_(14.5) = 10.88, *p* < 0.001. The two sets of attire were equated to be average In perceived strength (*M* = 4.35, *SD* = 0.52), *t*_*Welch*_(18.5) = 1.54, *p* = 0.14. However, due to confounds that are inherent in attire-based status antecedents^56^, the set of high-status (vs. low-status) attire was rated significantly higher in perceived trustworthiness (*M*_*high*_ = 5.22, *SD*_*high*_ = 0.61, *M*_*low*_ = 4.04, *SD*_*low*_ = 0.41), *t*_*Welch*_(22.7) = 6.05, *p* < 0.001, dominance (*M*_*high*_ = 4.88, *SD*_*high*_ = 0.80, *M*_*low*_ = 3.30, *SD*_*low*_ = 0.63), *t*_*Welch*_(24.6) = 5.77, *p* < 0.001, and attractiveness (*M*_*high*_ = 5.10, *SD*_*high*_ = 0.72, *M*_*low*_ = 4.11, *SD*_*low*_ = 0.55), *t*_*Welch*_(24.2) = 4.08, *p* < 0.001.

Using an open-source photo editing software^108^, we applied each of the 28 face stimuli to one low-status attire and one high-status attire. Each face was assigned to two different attires to facilitate counterbalancing. In other words, one set of faces was presented to approximately half of participants in low-status attire and the other set in high-status attire. For the other half of participants, the face sets wore high- and low-status attire, respectively. Each participant saw each of the 28 faces one time only. Although attires rather than colored frames were used as a proxy for status in this Experiment 2, participants were not told that the attires were meant to convey social status.

### Procedure

After the initial TurkPrime screening and consent form for the main study, participants completed the same 28-block Trust Game as in Experiment 1, this time using the attire-wearing face stimuli. Unlike in Experiment 1, participants did not complete any status-color association training because this training was not necessary—status was conveyed solely through attire in Experiment 2. After completing the Trust Game, participants rated the trustworthiness and social status of each of the 28 partners (exploratory). Participants first rated each of the 28 faces they interacted with during the trust game on trustworthiness using a scale of 1–7, with 7 being the most trustworthy. All faces were presented in random order unrelated to the order of the trust game task. Next, participants rated their perceptions of each face’s socioeconomic status (face-attire composites) using a scale of 1–7, with 7 being the highest status. All faces were presented in random order unrelated to the order of the trust game task. Following stimuli ratings, participants completed the same post-game measures from Experiment 1 (exploratory). Finally, participants were debriefed and given a completion code to receive compensation.

### Data analysis

Cumulative link mixed models for Experiment 2 were the same as in Experiment 1. We also again modeled the prior trial’s feedback as a main effect and interaction with the current partner’s status level (exploratory). Nonetheless, the primary effect of interest was the main effect of the current partner’s status level as defined by consensus ratings of the attire (confirmatory). In a second model, we used participant’s subjective rating of status to predict trust (exploratory). For the pre-registered linear mixed effects model, see Supplemental Materials S2.2.

## Experiment 3 Methods

Experiment 3 was pre-registered at https://osf.io/zevhb/. All data and materials are publicly available at https://osf.io/tcjw5/.

### Participants

To determine whether the experiment was sufficiently powered, we conducted a power analysis using a simulation-based approach for cumulative link mixed models that was adapted from a script for linear mixed models by Lane and Hennes^109^. We simulated 500 datasets based on variance parameters observed in Experiment 2 for the analysis relying on perceived social status. This simulation script is available for download at https://osf.io/tcjw5/. Simulations of several sample sizes suggested a sample of 350 participants would be adequately powered to detect a standardized main effect of perceived status as small as b = 0.10956 at 81.0% power (assuming a cumulative link mixed model; 77.4% power assuming a linear mixed model). To account for an exclusion rate of 12.3% (based on Experiment 2), we oversampled by 43 participants, up to 393.

A U.S.-based sample of 483 participants was recruited using Amazon Mechanical Turk. Using the same inclusion criteria as Experiment 2, participants were initially screened using TurkPrime to ensure their eligibility. As before, participants were given $2.00 USD for their participation and the opportunity for an additional $0–2 bonus payout. Two participants were excluded for failing to complete all or part of the Trust Game. To further ensure data quality, 107 participants who failed more than 2 out of 5 questions in the task instruction quiz were excluded from analysis. All remaining participants completed the full Trust Game and gave permission to use their data after debriefing. Lastly, in accordance with our pre-registration, we excluded from analysis 97 trials with response times slower than 10 s and 44 trials with response times faster than 75 ms. Following our a priori data exclusions, we were left with a sample of 374 participants (209 Female, 159 Male, 4 Non-binary, 1 Agender, 1 Femme Androgyne). Participant ages ranged from 18 to 34 years old (M = 28.7, SD = 3.71). All participants were based in the United States and self-reported proficiency in the English language. Two hundred forty-three (65.0%) participants identified as White/Euro American, 47 (12.6%) as Asian/Asian American, 38 (10.2%) as Black/African American, 27 (7.2%) as Latino/Hispanic American, 13 (3.5%) as Biracial/Multiracial, 5 (1.3%) Native American, and 1 (0.3%) Middle Eastern/Arab American. Participants’ subjective SES scores for themselves ranged from 1 (lowest possible SES) to 9 (second highest possible SES), with an average subjective SES score of 4.75 (SD = 1.60). This study is in accordance with relevant guidelines and regulations. Participants provided informed consent in accordance with the guidelines set by the Declaration of Helsinki, and the experimental protocol was approved by the University of Delaware’s Institutional Review Board.

#### Procedure

Up until the end of the Trust Game, stimuli, tasks, and procedures for Experiment 3 were the same as in Experiment 2. Following the Trust Game, participants rated each face on perceived trustworthiness, dominance, likeability (all exploratory), and perceived social status (confirmatory). Ratings for each dimension were collected in dimension-specific blocks that were randomized across participants. Participants then completed the same post-game demographics measures from Experiment 2. However, unlike in Experiment 2, we did not collect social value orientation because we found no effects and to limit task time.

### Ethics approval

Data files and analysis scripts for all analyses, including supplemental analyses, are available on Open Science Framework.

## Supplementary Information

Supplementary Information.

## Data Availability

All studies were pre-registered. All data files and analysis scripts for all analyses including supplemental analyses are available on the Open Science Framework.
