# Supplementary material for: Distrust as a form of inequality
Source: Sci Rep. 2023 Jun 19;13:9901. doi: 10.1038/s41598-023-36948-x (PMC10279663; doi:10.1038/s41598-023-36948-x)
Supplement: Supplementary file 1 — Supplementary Information. [file 41598_2023_36948_MOESM1_ESM.docx]

**SUPPLEMENTAL MATERIALS SECTIONS S1, S2 and S3**

**Distrust as a form of inequality**

# S1. Study 1 Supplemental materials

The first section of this supplement contains information relevant to the confirmatory analysis reported in the main text, as well as several of our pre-registered exploratory individual differences analyses for Study 1.

## S1.1 Methods and individual difference measures

***Power.***

**Study 1.** Because this was our first study to examine the effect of SES on trust decisions using a classic Trust Game, we estimated power for a 2 × 2 within-participants factorial design consisting of 147 participants (entered as a random grouping factor), and approximately 7 analyzable trials per condition. Due to our initial interest in both our confirmatory status hypothesis and exploratory hypotheses about previous partner return amount (i.e., feedback), we first ran a power analysis to examine power for a Status × Feedback interaction. Results suggested that a sample of 147 participants would be sufficiently powered to detect a significant Status × Feedback interaction at 80.4% power, assuming a small effect size of d = 0.144 and the default PANGEA variance parameters (var[error] = 0.333, var[participant*status*feedback] = 0.083). This same sample size would also have 80.1% power to detect a significant main effect of status, assuming a small effect size of d = 0.197 and the default PANGEA variance parameters (var[error] = 0.333, var[participant*status] = 0.167).

US-based participants between the ages of 18-40 were recruited via Mturk (n = 169). Participants were required to have at least a 70% approval rating on MTurk and were initially screened for eligibility via a brief demographics survey prior to the behavioral task. Each participant was paid $4.00 for completing the study, with an additional bonus varying between $0-2 USD depending on a randomly selected trial from the study. In line with our pre-registered exclusion criteria (https://osf.io/pge3c), one participant who failed 2 or more of 3 attention checks during the task was excluded. We also excluded 13 participants who failed at least 4 out of 5 questions on an initial instruction quiz that assessed whether participants understood the task. Lastly, to ensure participants were paying attention to the task, we excluded from analysis 44 trials with response times slower than 10 seconds and 14 trials with response times faster than 75 ms.

**Study 2.** To determine whether the experiment was sufficiently powered, we again conducted an a priori power analysis for linear mixed models using the PANGEA application (v0.2; Westfall, 2015). As this was the first study to examine the effect of partner socioeconomic status in the Trust Game using attire as the sole antecedent of status, the effect sizes and variances associated with the critical predicted interactions were not possible to predict a priori. Therefore, we used the default variance parameters in PANGEA (var[error] = 0.333, var[participant*status] = 0.167) to estimate power for the main effect of target status within a 2 (Status: low, high) × 2 (Partner Decision: keep, share) within-participants design consisting of 7 analyzable trials per condition.

***Stimuli.***

**Study 1.** The following inclusion criteria were used when selecting face images: (1) direct eye gaze, (2) upright head position, (3) no glasses, and (4) no piercings. Any jewelry or piercings were removed using Adobe Photoshop, where possible. Stimuli Cropped face images were then divided into four groups of seven faces that were equated, as confirmed by non-significant one-way ANOVAs, *F*(3,24) < 2.42, *p* > .09. The face set was perceived as White by 96.4% (*SD* = 3.5%) of Chicago Face Database raters. Faces were predominantly perceived as neutral in expression (*M* = 61.5%, *SD* = 17.0%), followed by angry (*M* = 17.9%, *SD* = 14.9%), sad (*M* = 12.2%, *SD* = 10.8%), and happy (*M* = 8.5%, *SD* = 14.1%). Based on a 7-point scale from 1 (Not at all) to 7 (Extremely), the face set was: (1) above-average in the intensity of their emotional expressions (*M* = 5.58, *SD* = 0.17), (2) average in perceived threat (*M* = 4.04, *SD* = 0.46) and dominance (*M* = 3.90, *SD* = 0.57), and (3) below-average in perceived attractiveness (*M* = 2.78, *SD* = 0.53), likeability (*M* = 3.34, *SD* = 0.35), and trustworthiness (*M* = 3.11, *SD* = 0.32).

**Study 2.** The images were split into four groups of seven faces that we equated, as confirmed by non-significant one-way ANOVAs, *F*(3,24) < 1.43, *p* > .25. The average face was perceived by Chicago Face Database raters to be approximately 26.7 years old (*SD* = 4.89 years). The set of 28 faces closely matched the average luminance of the Chicago Face Database (*M* = 159.98, *SD* = 12.10). Based on a 7-point scale from 1 (Not at all) to 7 (Extremely), the face set was: (1) average in perceived racial prototypicality (*M* = 3.49, *SD* = 0.78), and masculinity (*M* = 4.37, *SD* = 0.49), and (2) below-average in attractiveness (*M* = 2.99, *SD* = 0.54), dominance (*M* = 2.90, *SD* = 0.66), trustworthiness (*M* = 3.19, *SD* = 0.29), perceived threat (*M* = 2.43, *SD* = 0.59), neoteny (*M* = 2.46, *SD* = 0.58), sadness (*M* = 2.50, *SD* = 0.49), anger (*M* = 2.51, *SD* = 0.60), fear (*M* = 2.00, *SD* = 0.26), disgust (*M* = 2.14, *SD* = 0.42), unusualness (*M* = 2.55, *SD* = 0.54), surprise (*M* = 1.73, *SD* = 0.20), and happiness (*M* = 2.31, *SD* = 0.49).

***Subjective Status.***

Subjective socioeconomic status (SES) was assessed using the MacArthur Scale of Subjective Social Status (Adler et al., 2000). The MacArthur scale presents participants with a ladder comprising rungs labeled 1 to 10 in ascending order, where a 1 represents the rank of people who have the lowest standing amongst the general population in the U.S. and a 10 represents the rank of people who have the highest standing among the general population of the U.S. Below, we include th­­e question text that participants were shown:

| 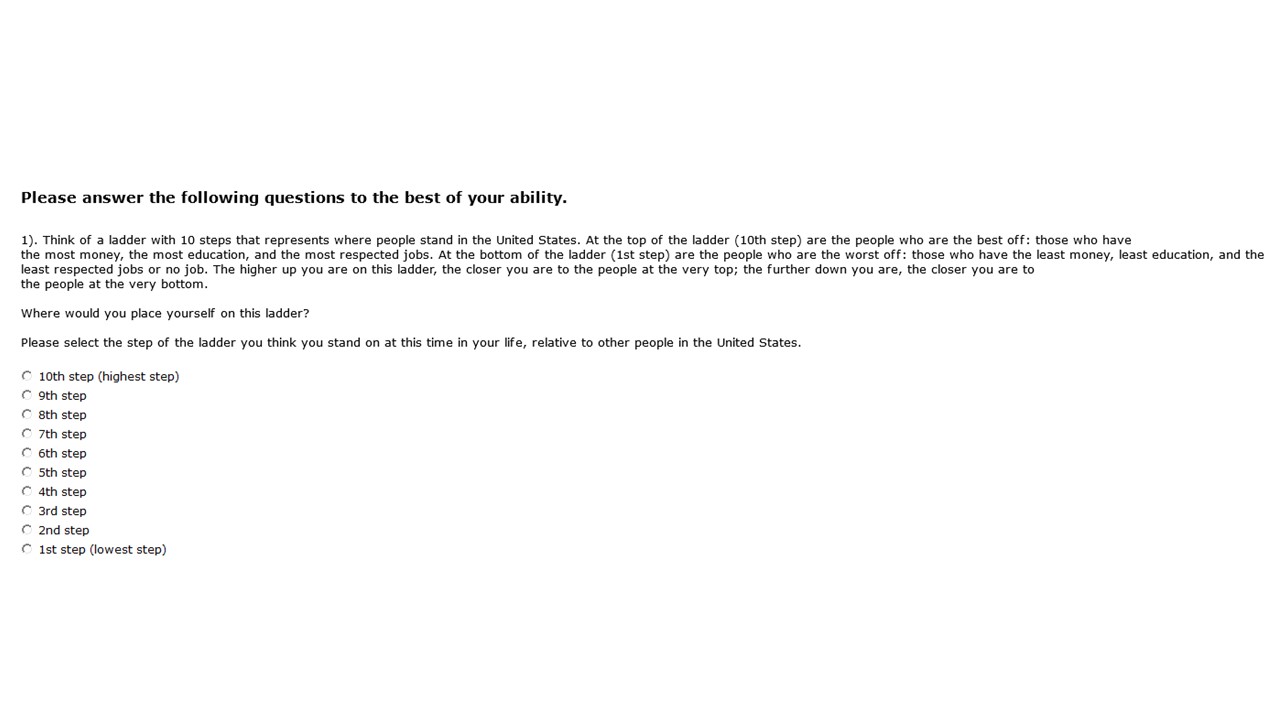 |
| --- |
| Figure S1. Subjective Status Questionnaire |

***Objective Status.***

Following previous recommendations (Oakes & Rossi, 2003) and other work on how perceived social status affects decision making (Mattan et al., 2020), we measured a number of single-item measures that reflect income, education, and assets. These measures were used to compute a composite score of objective SES that equally weights income, education, and assets. Below we include the question text that participants were shown:

| 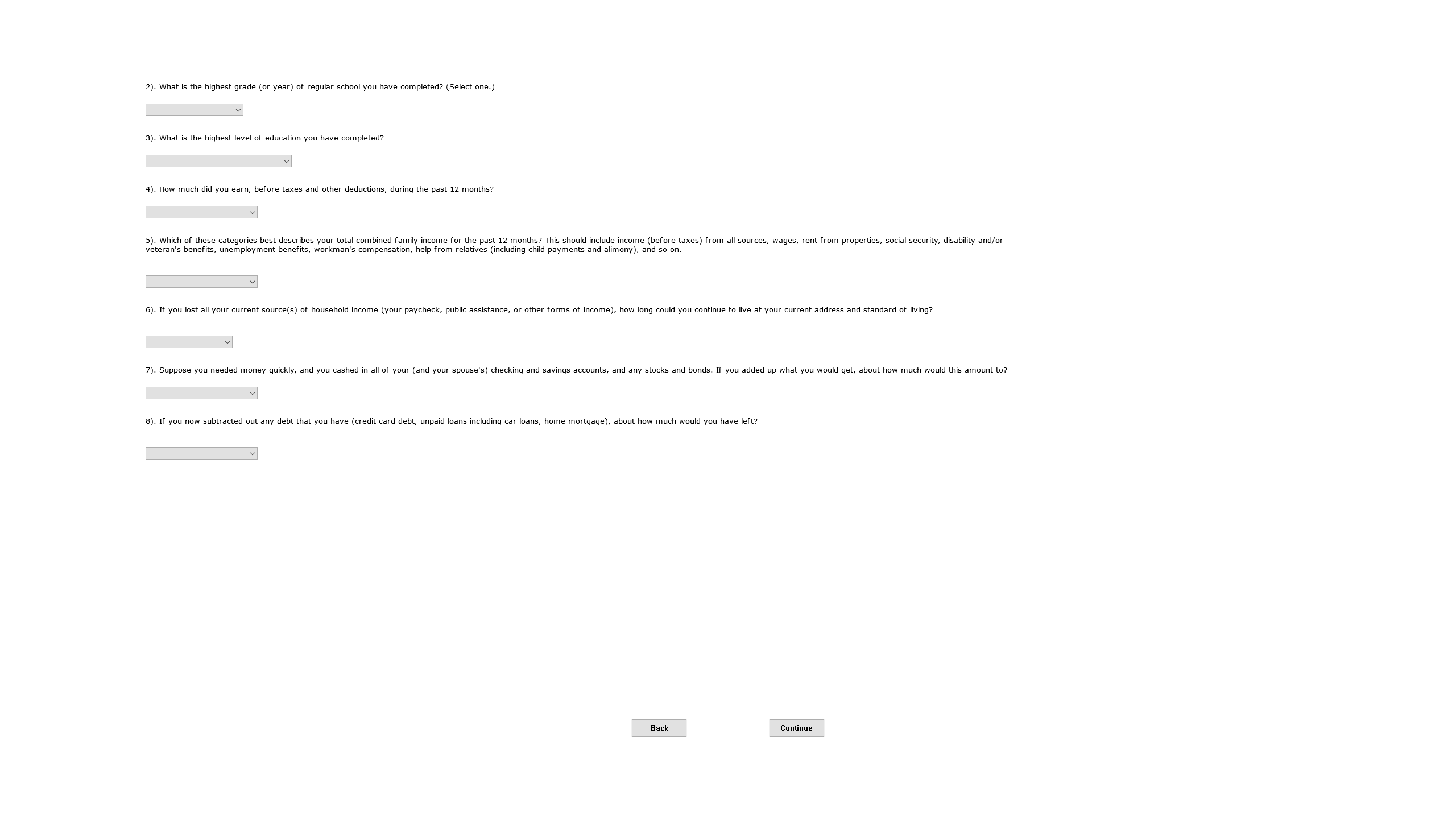 |
| --- |
| Figure S2. Objective Status Questionnaire |

***Social Value Orientation.***

Social value orientation was measured through the 12-item Delaware adaptation of Liebrand’s Ring Measure^1,2^. The measure has 12 items, each of which is a unique resource allocation choice. Participants are told that they are randomly paired with another partner, and that they will make choices that can produce points for or take points away from themselves and their partner. Each of the 12 items has a different combination of two outcomes that the participant must choose between. By utilizing each of the 12 choices that the participant has made, a categorical measure of social value orientation was computed for each person.

While we collected this questionnaire for both Studies 1 and 2, we did not analyze trust decisions as a function of SVO responses.

### ***Perceptions of Trust***

Across all three studies, we measured the extent to which participants felt that high versus low status partners shared at different rates.

## ***S1.2 Supplementary analyses***

Below we summarize findings from our individual differences analyses. To avoid redundancy with the main text, we only report main effects or interactions that include the specific individual difference measure within each section.

### ***Partner Feedback Predicting Trust***

#### LMER Results

Results yielded a significant main effect of partner status on trust decisions, *b* = 0.428, *SE* = 0.136, *CI_95%_* = [0.160 0.695], *t*(154.465) = 3.133, *p* = 0.002. However, there was no significant main effect of partner feedback predicting trust, *p* = 0.362. The interaction of partner feedback and current partner’s status was non-significant, *p* = 0.064.

### ***Subjective SES***

#### **CLMM Results**

Results yielded no significant main effect of participant’s subjective SES predicting trust, *p* = 0.148. Additionally, the interaction of subjective SES and current partner’s status was non-significant, *p* = 0.380.

#### **LMER Results**

Results yielded no significant main effect of participant’s subjective SES predicting trust, *p* = 0.167. Additionally, the interaction of subjective SES and current partner’s status was non-significant, *p* = 0.938.

### ***Objective SES***

#### **CLMM Results**

Results yielded no significant main effect of partner feedback predicting trust, *p* = 0.562. The interaction of partner feedback and current partner’s status was non-significant, *p* = 0.683.

#### **LMER Results**

Results yielded no significant main effect of partner feedback predicting trust, *p* = 0.590. The interaction of partner feedback and current partner’s status was non-significant, *p* = 0.472.

### ***S1.3 LMER Model and Random Effects Determination***

Below we list steps we took to determine random effects for all LMER models. These parameters were employed across all 3 studies for each of the LMER models. While our final analyses used the CLMM function in R, we offer our original pre-registered steps for random effects determination for the LMER models.

As far as allowed by these data, we modeled a correlated random intercept and random slope for Partner Status Level. For the full models (i.e., the initial regression models), high status was contrast coded as 0.5, and low status was coded as -0.5.

Data were analyzed with the following model:

- omnibus.model_1=lmer(MoneyTrusted ~ PartnerStatusLevel + (1 + PartnerStatusLevel| Subject), data=data)

In other words, these models tested the main effect of partner status (IV) for the dependent variable: money trusted. The lme4 package^3^ with lmerTest^4^ in the R programming language^5^ was used to run the analyses.

Determination of random-effects structures: To the extent possible, we allowed for between-participants variance in intercepts and slopes for all within-subject factors (i.e., random effects) and the correlations between these random effects in confirmatory and exploratory analyses. However, sometimes, full models failed to converge or are in fact over-fitted^3^. If the full model failed to converge or was over-fitted, we followed the steps outlined below in the order specified. If the full model converged and was not over-fitted (as determined by the PCA procedure detailed below), we used the full model as our final model.

In the event of convergence failure and/or an over-fitted full model:

1) We increased the max iterations allowed for convergence (up to 100,000) and reset the optimizer used for convergence.

2) We removed all correlation parameters from the random effects structure, ran this model, and conducted a PCA on the resulting model to determine how many random effects can be supported by these data, as detailed by Bates and colleagues at https://arxiv.org/pdf/1506.04967v1.pdf.

3) Based on the number of redundant dimensions in these data (determined in the previous step), we removed slopes from the random effects structure, starting with random slopes corresponding to higher order interactions. When choosing between random effects for interactions on the same order (e.g., two two-way interactions), removed the random slopes that account for the least amount of variance in the model from the previous step.

4) We re-ran the PCA on the reduced model for step 3 to ensure that the model is not overfitted. If the reduced model remained over-fitted (i.e., contained components that account for exactly zero variance), we removed random slopes and repeated the PCA until all components account for some non-zero amount of variance.

5) Next, we added in all possible correlation parameters, running PCA to avoid over-fitting the model.

a) If adding even one correlation parameter reduced the dimensionality of these data, then we did not add any correlation parameters. Use the final model from step 4.

b) If it was possible to include some but not all correlation parameters without reducing the dimensionality of these data, we prioritized conserving the largest correlation parameters and excluding any correlation parameters that were equal to 1, 0, or NaN). The final resulting model from step 5 with added correlation parameters had to result in a significant log-likelihood ratio test when compared to the final reduced model from step 4. Otherwise, we used the simpler model resulting from step 4.

c) If the dimensionality of these data was conserved after including all possible correlation parameters and the model with all possible correlation parameters resulted in a significant log-likelihood ratio test when compared with the final reduced model from step 4, then we pruned the correlation parameters equal to 0, 1, or NaN. If all correlation parameters fell between 0 and 1, then we attempted to prune the smallest correlation parameters from the model. However, the pruned model had to result in a non-significant log-likelihood ratio test compared to the model with all possible correlation parameters (see the start of step 5) and a significant log-likelihood ratio test compared to the model without any correlation parameters (see the end of step 4). If these conditions were not met, we did not prune any correlation parameters from the model.

d) If the dimensionality of these data was conserved after including all possible correlation parameters but the model with all possible correlation parameters resulted in a non-significant log-likelihood ratio test when compared with the final reduced model from step 4, then we used the simpler model resulting from step 4.

### ***Random-effects determination code***

#CLMM

modOrdStudy1a <- clmm(response ~ currstat + (1+currstat|subject), data=tgdata1, Hess=TRUE)

#Failed to converge, use intercept only model

modOrdStudy1b <- clmm(response ~ currstat + (1|subject), data=tgdata1, Hess=TRUE)

tgdata1$response<- as.numeric(tgdata1$response)

#LMER

modOrdStudySSLM1a <- lmer(response ~ currstat +(1+currstat|subject) , data=tgdata1, control=lmerControl(optimizer="bobyqa", optCtrl=list(maxfun=100000)))

summary(modOrdStudySSLM1a)

# #Model Converges

# S2. Study 2 Supplemental materials

The second section of this supplement contain information relevant to the confirmatory analysis reported in the main text, as well as several of our pre-registered exploratory individual differences analyses for Study 2.

## ***S2.1*** Individual difference measures

In Study 2, we used identical questionnaires to assess our participant’s objective and subjective SES, explicit perceptions of trust and social value orientation as we did in Study 1.

## ***S2.2 Supplementary analyses***

### CLMM Models

#### Subjective SES Analysis

Results failed to yield any additional significant main effects or interactions compared to the omnibus model, *p* > 0.231.

#### Objective Status Analysis

Results failed to yield any additional significant main effects or interactions, *p* > 0.144.

#### Subjective Status as a function of Partner Feedback

Results revealed that perceived partner status robustly predicted greater perceptions of trust, such that partners greater perceptions of subjective social status predicted greater dollar amounts shared, effectively mirroring the patterns of greater perceptions of partner social status on dollar amounts trusted in the main text, *b* = 0.961, *SE* = 0.041, *CI_95%_* = [0.881 1.041], *z*(8) = 23.46, *p* < .001.

### LMER Results

#### Omnibus Model

Results revealed that partner feedback robustly predicted greater perceptions of trust, such that partners who kept money were viewed as significantly more trustworthy compared to those who shared back the money trusted to them, *b* = -0.248, *SE* = 0.113, *CI_95%_* = [-0.234 -0.014], *t*(105) = -2.216, *p* = 0.029. No significant differences in trust decisions were observed as a function of consensus ratings of partner status, *p* = 0.356. The interaction of current partner status and partner feedback failed to reach significance, *p* = 0.390.

#### Subjective Status Analysis

Results failed to yield any additional significant main effects or interactions, *p* > 0.231.

#### Objective Status Analysis

Results failed to yield any additional significant main effects or interactions, *p* > 0.253.

## ***S2.3 Random Effects Determination***

Study 2 used identical procedures to determine random effects in Study 1.

### ***Random-effects determination code***

#CLMM

###Confirmatory omnibus model####

modOrdStudy2 <- clmm(response ~ currstat*prevfb + (1+currstat*prevfb|subject), data=tgdata2, Hess=TRUE)

# converges

###Exploratory omnibus model####

modOrdStudy2 <- clmm(response ~ INDStatus + (1+INDStatus|subject), data=tgdata2, Hess=TRUE)

# full model converges

tgdata2$response<- as.numeric(tgdata2$response)

#LMER

#Confirmatory Omnibus model

modOrdStudySSLM2a <- lmer(response ~ currstat*prevfb + (1+currstat*prevfb|subject), data=tgdata2, control=lmerControl(optimizer="bobyqa", optCtrl=list(maxfun=100000)))

#Model failed to converge

modOrdStudySSLM2b <- lmer(response ~ currstat*prevfb + (1+currstat*prevfb||subject), data=tgdata2, control=lmerControl(optimizer="bobyqa", optCtrl=list(maxfun=100000)))

# Model still failed to converge. Removing 2 way interaction

modOrdStudySSLM2c <- lmer(response ~ currstat*prevfb + (1+currstat+prevfb||subject), data=tgdata2, control=lmerControl(optimizer="bobyqa", optCtrl=list(maxfun=100000)))

rePCA(modOrdStudySSLM2c)

#Converges and parameters are not near 0 or 1

modOrdStudySSLM2d <- lmer(response ~ currstat*prevfb + (0+currstat+prevfb||subject)+ (1|subject) , data=tgdata2, control=lmerControl(optimizer="bobyqa", optCtrl=list(maxfun=100000)))

modOrdStudySSLM2e <- lmer(response ~ currstat*prevfb + (1+currstat||subject)+ (0+prevfb |subject) , data=tgdata2, control=lmerControl(optimizer="bobyqa", optCtrl=list(maxfun=100000)))

modOrdStudySSLM2f <- lmer(response ~ currstat*prevfb + (1+prevfb||subject)+ (1+currstat|subject) , data=tgdata2, control=lmerControl(optimizer="bobyqa", optCtrl=list(maxfun=100000)))

anova(modOrdStudySSLM2d,modOrdStudySSLM2e)

#non-significant, use D

anova(modOrdStudySSLM2d,modOrdStudySSLM2f)

#non-significant, use D

modOrdStudySSLM2g <- lmer(response ~ currstat*prevfb + (0+currstat||subject)+ (1+prevfb|subject) , data=tgdata2, control=lmerControl(optimizer="bobyqa", optCtrl=list(maxfun=100000)))

modOrdStudySSLM2h <- lmer(response ~ currstat*prevfb + (0+prevfb||subject)+ (1+currstat|subject) , data=tgdata2, control=lmerControl(optimizer="bobyqa", optCtrl=list(maxfun=100000)))

anova(modOrdStudySSLM2g,modOrdStudySSLM2h)

#Use g

modOrdStudySSLM2I <- lmer(response ~ currstat*prevfb + (1+prevfb+currstat|subject) , data=tgdata2, control=lmerControl(optimizer="bobyqa", optCtrl=list(maxfun=100000)))

#converges

#Testing the most optimal RFX structure

anova(modOrdStudySSLM2I,modOrdStudySSLM2g)

#non-significant use simpler model (g)

anova(modOrdStudySSLM2g,modOrdStudySSLM2d)

#non-significant use simpler model (d)

anova(modOrdStudySSLM2d,modOrdStudySSLM2c)

#non-significant use simpler model (c)

modOrdStudySSLM2c <- lmer(response ~ currstat*prevfb + (1+currstat+prevfb||subject), data=tgdata2, control=lmerControl(optimizer="bobyqa", optCtrl=list(maxfun=100000)))

#Final LMER model

# ***S3. Study 3 Supplemental materials***

The third section of this supplement contains information relevant to the confirmatory analysis reported in the main text, as well as several of our pre-registered exploratory individual differences analyses for Study 3.

## ***S3.1*** Individual difference measures

In Study 3, we used identical questionnaires to assess our participant’s objective and subjective SES as well as explicit perceptions of trust as we did in the previous two studies. We did not collect participant’s social value orientations in Study 3.

## ***S3.2 Supplementary analyses***

### LMER Results

#### Omnibus Model LMER Results

Results revealed that perceived partner status robustly predicted greater perceptions of trust, such that perceived high-status partners were viewed as significantly more trustworthy compared to perceived low-status partners, *b* = 0.146, *SE* = 0.030, *CI_95%_* = [0.088 0.206], *t*(336) = 4.869, *p* < 0.001.

### CLMM models testing the impact of perceived partner traits

#### Trust as a function of Partner Feedback

Results revealed that partner feedback robustly predicted greater perceptions of trust, such that partners who shared were viewed as significantly more trustworthy compared to those who kept the money shared *b* = 0.157, *SE* = 0.040, *CI_95%_* = [0.079 0.235], *z* = 3.957, *p* < .001.

#### Subjective Status as a function of Partner Feedback

Results found that perceived partner social status did not differ as a function of partner feedback, *p* = 0.735.

#### Perceived dominance as a function of Partner Feedback

Results found that perceived partner dominance did not differ as a function of partner feedback, *p* = 0.913.

#### Perceived likeability as a function of Partner Feedback

Results revealed that partner feedback robustly predicted greater perceptions of partner likeability, such that partners who shared were rated as significantly more likeable than partners who kept the money trusted to them, *b* = 0.096, SE = 0.040, *CI_95%_* = [0.0186 0.174], *z*(8) = 2.430, *p* =.015.

#### Trust as a function of Partner Feedback

Results yielded no significant main effect of partner feedback predicting trust decisions, *p* = 0.181. Additionally, the interaction of partner feedback and perceived partner’s status was non-significant, *p* = 0.627.

#### Trust decisions, consensus ratings of partner status and partner feedback

Results yielded no significant main effect of ascribed partner status predicting trust decisions, *p* = 0.147. Additionally, the interaction of partner feedback and consensus ratings of partner status was non-significant, *p* = 0.183.

#### Objective Status Analysis

Results yielded no significant main effect of participant’s objective SES predicting trust, *p* = 0.764. Additionally, the interaction of objective SES and current partner’s perceived status was non-significant, *p* = 0.350.

#### Subjective Trust Ratings Analysis

Results yielded no significant main effect of participant’s subjective SES predicting trust, *p* = 0.307. Additionally, the interaction of subjective SES and current partner’s perceived status was non-significant, *p* = 0.847.

## ***S3.3 CLMM Random Effects Determination***

Determination of random-effects structures: To the extent possible, we allowed for between-participants variance in intercepts and slopes for all within-subject factors (i.e., random effects) and the correlations between random effects. However, sometimes, full models failed to converge or were in fact over-fitted^6^ (Bates et al., in preparation:

https://arxiv.org/pdf/1506.04967v1.pdf). If the full model failed to converge or was over-fitted, we followed the steps outlined below in the order specified. If the full model converged and did not lead to any singular fits, then we used the full model for our final model. In the event of convergence failure and/or a singular fit in the model1, we:

1) Increased the max iterations allowed for convergence (maxIter = 10000000, maxLineIter = 10000000).

2) Reset the optimizer used for convergence (method = "nlminb").

3) Removed all correlation parameters from the random effects structures (i.e., by participant and by stimulus) to see whether any convergence issues or singular fits arise.

4) If errors or warnings were still observed after the preceding step, we iteratively removed slopes from the random effects structures, starting first with any slopes under the stimulus grouping factor before removing any slopes under the participant grouping factor. Within each grouping factor, we started by removing random slopes that corresponded to higher order interactions. When choosing between random effects for interactions on the same order (e.g., two two-way interactions), we removed the random slope that accounted for the least amount of variance in the model from the previous step.

5) Once a model was found that converged, we added in all possible correlation parameters back into the model.

a) If adding correlation parameters produced errors or singular fit warnings, then we did not add any correlation parameters. Use the final model from step 4.

c) If the model with correlation parameters successfully converged without singular fits and that model resulted in a significant log-likelihood ratio test when compared with the final reduced model from step 4, then the model with correlation parameters became the final model.

d) If the model with correlation parameters successfully converged without singular fits but that model resulted in a non-significant log-likelihood ratio test when compared with the final reduced model from step 4, then we used the simpler model resulting from step 4.

### ***Random-effects determination code***

modOrdStudy3a <- clmm(response ~ INDStatus + (1+INDStatus|subject), data=tgdata3, Hess=TRUE)

#model failed to converge. Use intercept-only model

modOrdStudy3b <- clmm(response ~ INDStatus + (1|subject), data=tgdata3, Hess=TRUE)

####LMM random effects####

tgdata3$response=as.numeric(tgdata3$response)

modStudy3LMMa <- lmer(response ~ INDStatus + (1+INDStatus|subject), data=tgdata3, control=lmerControl(optimizer="bobyqa", optCtrl=list(maxfun=100000)))

modStudy3LMMb <- lmer(response ~ INDStatus + (1|subject), data=tgdata3, control=lmerControl(optimizer="bobyqa", optCtrl=list(maxfun=100000)))

anova(modStudy3LMMa,modStudy3LMMb)

#Significant, use more complex model

References

1. Karagonlar, G. & Kuhlman, D. M. The role of social value orientation in response to an unfair offer in the ultimatum game. *Organizational Behavior and Human Decision Processes* **120**, 228–239 (2013).

2. Liebrand, W. B. The effect of social motives, communication and group size on behaviour in an N‐person multi‐stage mixed‐motive game. *European Journal of Social Psychology* **14**, 239–264 (1984).

3. Bates, D., Mächler, M., Bolker, B. M. & Walker, S. C. Fitting linear mixed-effects models using lme4. *Journal of Statistical Software* **67**, (2015).

4. Kuznetsova, A., Brockhoff, P. B. & Christensen, R. H. B. lmerTest Package: Tests in Linear Mixed Effects Models. *Journal of Statistical Software* **82**, 1–26 (2017).

5. R Core Team. R: A language and environment for statistical computing. (2017).

6. Bates, D., Kliegl, R., Vasishth, S. & Baayen, H. Parsimonious Mixed Models. *arXiv* **1506**, (2015).
